# *Drosophila* Reporter Vectors Compatible with ΦC31 Integrase Transgenesis Techniques and Their Use to Generate New Notch Reporter Fly Lines

**DOI:** 10.1534/g3.111.001321

**Published:** 2012-01-01

**Authors:** Ben E. Housden, Kat Millen, Sarah J. Bray

**Affiliations:** Department of Physiology, Development and Neuroscience, University of Cambridge, Cambridge CB2 3DY, United Kingdom

**Keywords:** reporter plasmids, ΦC31 integrase, fluorescent protein, Drosophila, Notch

## Abstract

Complex spatial and temporal regulation of gene activity is fundamental to development and homeostasis. The ability to decipher the DNA sequences that accurately coordinate gene expression is, therefore, of primary importance. One way to assess the functions of DNA elements entails their fusion to fluorescent reporter genes. This powerful approach makes it possible to visualize their regulatory capabilities when reintroduced into the developing animal. Transgenic studies in *Drosophila* have recently advanced with the introduction of site-specific, ΦC31 integrase–mediated approaches. However, most existing *Drosophila* reporter vectors are not compatible with this new approach and have become obsolete. Here we describe a new series of fluorescent reporter vectors optimized for use with ΦC31 transgenesis. By using these vectors to generate a set of Notch reporter fly lines, we demonstrate their efficacy in reporting the function of gene regulatory elements.

Understanding the mechanisms employed by regulatory DNA sequences to control gene expression is essential to the study of development and disease. Tools enabling this are, therefore, of major importance. One method for deciphering genomic regulatory information utilizes *in vivo* reporter assays to assess the activity of putative enhancers. For such analysis, the DNA is subcloned adjacent to easily monitored reporter genes, such as encoding β-galactosidase or green fluorescent protein (GFP), in vectors designed for transgenesis. This approach has been widely exploited in *Drosophila*, where stably inherited, single-copy transgene insertions could be generated with p-element transposon-based vectors (Rubin and Spradling 1982; Spradling and Rubin 1982). With this method, transgenes are inserted randomly in the genome, with a preference for promoter regions (Bellen *et al.* 2004). Expression from such insertions is often influenced by the surrounding sequences (position effects), leading to difficulties in interpreting patterns generated.

More recently, a transformation system has been introduced which exploits the integration mechanism used by bacteriophage ΦC31 (Groth *et al.* 2004). A phage integrase induces recombination between *attP* (phage genome) and *attB* (bacterial genome) sequences (Groth and Calos 2004; Thorpe and Smith 1998). Several groups have established transgenic fly lines containing *attP* sites (platforms) at specific, nonmutagenic locations (Bateman *et al.* 2006; Bischof *et al.* 2007; Groth *et al.* 2004; Markstein *et al.* 2008; Venken *et al.* 2006). Injection of *attB*-containing vectors with a source of ΦC31 integrase results in integration of the vector into the genome at the *attP* platform.

The ΦC31 system is more efficient than previous techniques, and as integration occurs at specific *attP* sites, insertions are directly comparable and mapping is unnecessary. However, most vectors for *in vivo* reporter assays, lacking *attB* sites, are incompatible with this method. Two adapted vectors with *attB* sequences have recently been made, but both use Gateway cloning and one retains p-element ends, precluding subsequent use of p-element mutagenesis in the flies generated (Aerts *et al.* 2010; Boy *et al.* 2010). We have generated a new series of compatible vectors that contain no unneccessary sequences and are optimized for enhancer detection due to the position of the cloning site, inclusion of insulators, and use of multiple reporters.

To achieve this, we adapted elements from the existing *HZ50PL-lacZ* enhancer–detecting vector (Hiromi and Gehring 1987) and a high copy p-element transformation plasmid [p-WhiteRabbit; Dunin-Borkowski and Brown (1995)]. We combined the *hsp70* minimal promoter from *HZ50PL-lacZ* with *eGFP*, *mCherry*, *lacZ*, or *venus[PEST]-YFP* coding sequences. Incorporating these reporters into a plasmid containing the p-WhiteRabbit vector backbone, *mini-white*, and kanamycin resistance genes (*kan*) in combination with an *attB* sequence enables use of the ΦC31 system. A *lox-p* site was included to allow removal of *kan* and platform sequences after genomic integration.

To minimize influence from position effects, the *mini-white* gene and vector backbone are arranged to flank the reporter gene after integration (Figure 1). We also inserted insulator (*gypsy*) elements, which have been shown to be effective in reducing the influence of neighboring sequences, flanking *mini-white* and the reporter gene (Barolo *et al.* 2000; Barolo *et al.* 2004) (Figure 1, purple circles). Resulting vectors are named after the originating plasmid (pWhiteRabbit), substituting the color prefix according to the type of reporter (pGreenRabbit, etc.). These vectors are compatible with a wide range of experiments, including live imaging. For example, destabilized Venus[PEST]-YFP could be used when perdurance of the reporter would be an issue or when fine-scale temporal differences in expression are investigated (Aulehla *et al.* 2008; Nagai *et al.* 2002). Furthermore, the different reporters enable several regulatory elements to be analyzed simultaneously.

**Figure 1:**
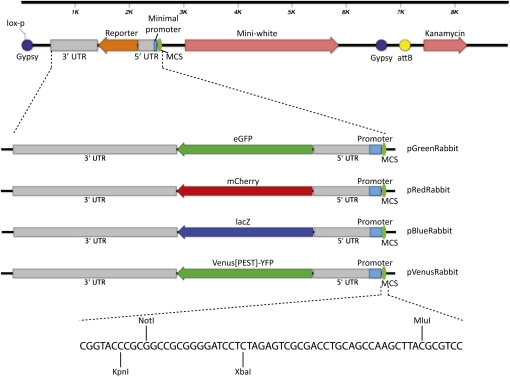
Reporter vectors compatible with ΦC31 transgenesis techniques. Diagram of vector backbone (top) into which four different reporters have been inserted as indicated. All vectors carry kanamycin resistance and use *mini-white* as a transformation marker. All reporters are downstream of a minimal *hsp70* promoter and are flanked by UTRs from the *hsp70* gene. The positions of *gypsy* (SuHw) insulator sequences (purple), *lox-p*, *attB* (yellow), and multiple cloning site (MCS) unique restriction sites are indicated.

## Reporters show no basal expression

**Figure 2:**
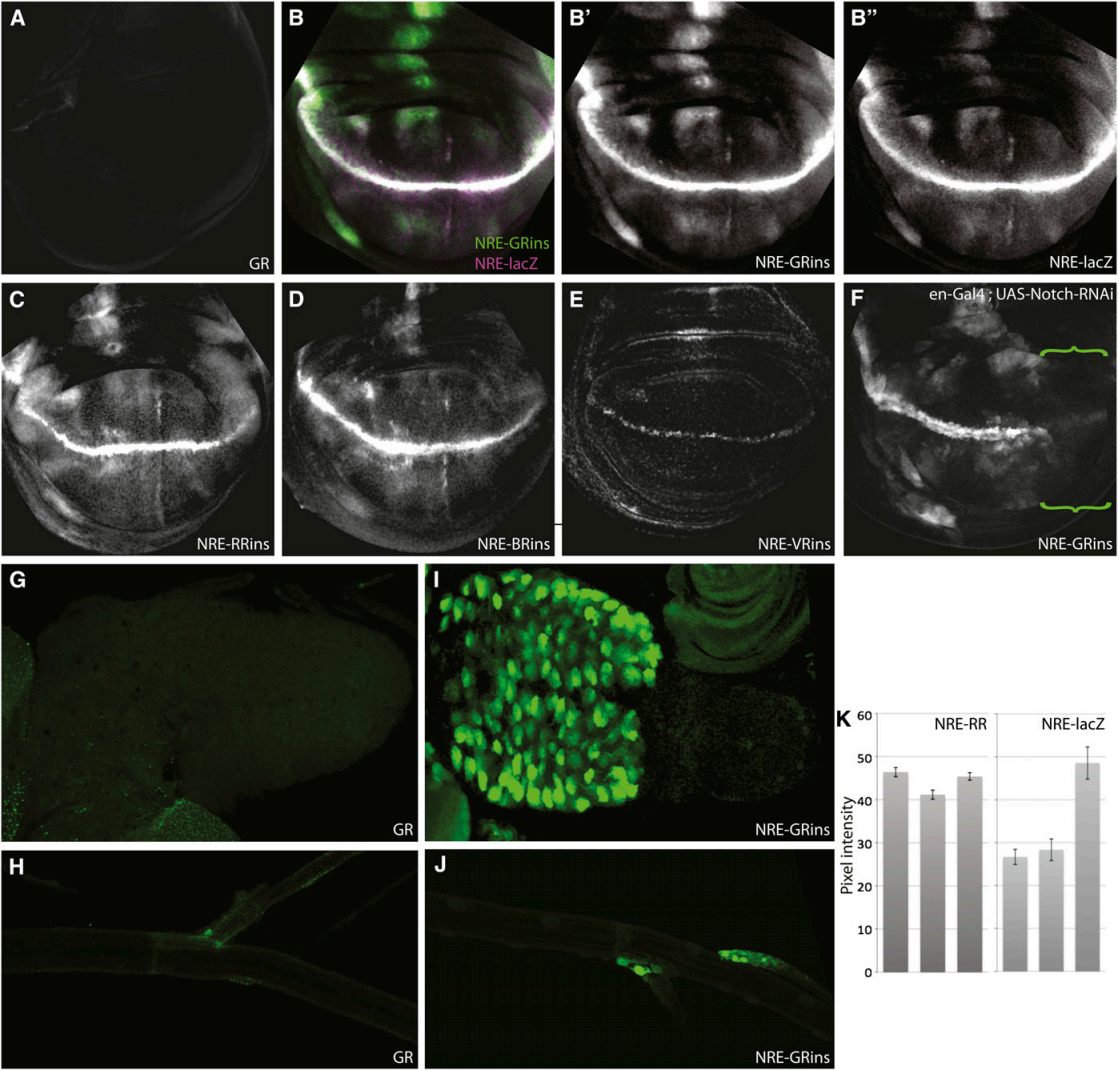
Vectors accurately report expression pattern from a Notch responsive element. (A) Basal expression from pGreenRabbit (GR) integrated at platform 51D. (B–E) Expression from indicated reporter vectors driven by the Notch responsive element (NRE) in the wing pouch of third instar larvae. *NRE-lacZ* (B, magenta; B′′, white) is a previously reported Notch reporter (Furriols and Bray 2001). The same NRE was used to generate *NRE-GreenRabbit* (NRE-GRins; B, green; B′, white); *NRE-RedRabbit* (NRE-RRins; C); *NRE-BlueRabbit* (NRE-BRins; D); and *NRE-VenusRabbit* (NRE-VRins; E). *NRE-VRins* was imaged with 10× excitation. (F) *Notch-RNAi* expression in the posterior compartment (green braces; driven using *en-Gal4*) eliminates expression from *NRE-GRins*. (G, H) Basal expression from pGR integrated at platform 51D in the larval brain (G) and trachea (H). (I, J) Expression from *NRE-GRins* in the larval brain (I) and trachea (J). Suffix “ins” indicates that the constructs contained insulators. (K) Comparison of expression levels from independent transgenic lines (average pixel intensity in the wing pouch measured using ImageJ). *NRE-RR* insertions at the same platform (86Fb) produce similar expression levels. *NRE-lacZ* insertions generated using p-element transgenesis are expressed at varying levels. At least five discs were quantified per genotype. Error bars show standard error of the mean. Primary antibodies were rabbit α-GFP (Molecular Probes, 1/500) (A, B, and E–J); rabbit α-dsRed (ClonTech, 1/50) (C); and mouse α-βGalactosidase (Developmental Studies Hybridoma Bank, 1/20) (B and D).

One important criterion for reporter constructs is that basal expression levels should be low. We tested whether pGreenRabbit gave any expression in the absence of an enhancer by generating insertions in several *attP* platform lines (2A, 22A, 51D, 68E, 81C, and 96E). In no case was GFP expression detected in the wing disc (Figure 2A and data not shown). Similarly, no basal expression could be detected in larval brains or trachea, confirming their efficacy as enhancer-detection vectors (Figure 2, G and H).

## Vectors accurately report Notch responsive enhancer activity

To test the functionality of these vectors, we inserted a previously characterized Notch responsive element (NRE) (Furriols and Bray 2001). Costaining to detect expression from *NRE-GreenRabbit* and the previous *NRE-lacZ* (Figure 2, B-B”) revealed identical patterns in wing discs. Furthermore, *NRE-GreenRabbit* gave the expected expression patterns elsewhere. In trachea, GFP was detected only in nests of cells at the tracheal branch points, as reported for the parent NRE reporter (Furriols and Bray 2001), and in larval brains, it was present in imaginal neuroblasts, a known site of Notch activity (Almeida and Bray 2005) (Figure 2, I and J). Notch responsiveness of *NRE-GreenRabbit* was confirmed by expressing *Notch-RNAi* in posterior compartments of wing discs. Under these conditions, expression was lost, indicating that it is dependent on Notch signaling (Figure 2F). NRE function was also accurately reported with mCherry, lacZ, and Venus[PEST]-YFP variants (Figure 2, C-E). As expected, independent insertions of *NRE-RedRabbit* at a single platform site gave reproducible expression levels compared with independent *NRE-lacZ* p-element insertions (Figure 2K). The new vectors, therefore, accurately and reproducibly report known expression patterns from enhancer elements. The lines generated will also provide useful tools for analysis of Notch pathway activity *in vivo*.

## Transgenes are resistant to position effects

**Figure 3:**
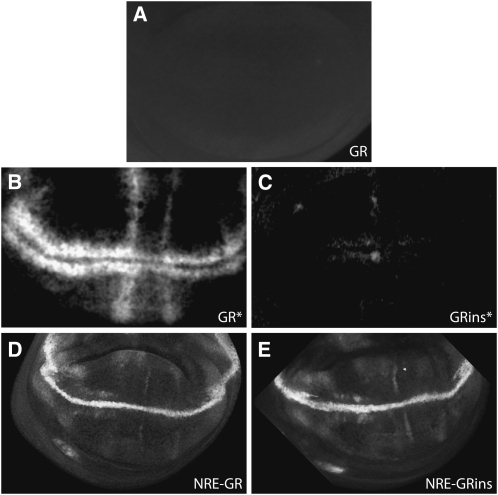
Insulators and buffering sequences are effective in preventing position effects without interfering with local enhancer-driven expression. Expression from pGreenRabbit in the absence of an enhancer sequence under the following conditions: buffering sequences present without insulators (GR; A); buffering sequences removed by Cre-induced recombination between *lox-p* sites without insulators (GR*; B); and buffering sequences removed with insulators (GRins*; C). Expression driven by the NRE enhancer in the absence (NRE-GR; D) or presence (NRE-Grins; E) of insulators. (A) and (C) were imaged using 4× higher excitation compared with other images. Primary antibody was rabbit α-GFP (Molecular Probes, 1/500) for all images.

To test susceptibility to surrounding sequences, we analyzed expression from pGreenRabbit at a location prone to position effects from a neighboring gene (51D) (http://flyc31.frontiers-in-genetics.org/). No expression was detected in wing imaginal discs (Figure 3A) or in several other tissues (Figure 2, G and H), indicating that the flanking vector sequences are effective buffers. When these vectors were removed by inducing recombination between *lox-p* sites, patterned reporter expression was present but greatly attenuated in the presence of insulators (Figure 3, B-C). Therefore, both the buffering sequences and insulators are effective in preventing position effects and make the vectors resistant to influences from surrounding DNA. Furthermore, the insulator sequences have no adverse effects on vector function, (Figure 3, D and E).

In summary, we have constructed a series of four reporter vectors specifically designed for use with ΦC31-mediated transgenesis that enable analysis and direct comparisons of different enhancers. Insulator elements and buffering sequences have been incorporated to protect the reporter gene from position effects. Using these vectors, we have produced a new generation of Notch reporter flies. These fly-lines demonstrate that the transgenes are effective reporters of enhancer-driven expression and, therefore, that the vectors constitute a flexible set of tools for *in vivo* enhancer assays in *Drosophila*.
